# Doyle silicone splint insertion: endoscopy-assisted versus nasal speculum assisted

**DOI:** 10.1016/j.bjorl.2020.07.005

**Published:** 2020-08-19

**Authors:** Secil Bahar Dal

**Affiliations:** VKV Amerikan Hastanesi, Otolaryngology Department, Istanbul, Turkey

**Keywords:** Endoscopic sinus surgery, Septoplasty, Middle turbinate, Lateralization, Nasal splint

## Abstract

**Introduction:**

Septoplasty and endoscopic sinus surgery are very often concurrently performed operations in otolaryngology practice. The most common complication of endoscopic sinus surgery is lateralization of the middle turbinate. In our practice intranasal stenting is done routinely with Doyle silicone splints.

**Objective:**

Retrospectively, we aimed to review the postoperative period and to document efficacy of endoscopy-assisted Doyle silicone splint application on prevention of middle turbinate lateralization.

**Methods:**

Patients who had failed medical therapy and who underwent elective primary *endoscopic sinus surgery* for chronic rhinosinusitis with septal deviation requiring septoplasty were included to the study. Doyle silicone splints were inserted to all patients at the end of the operation with nasal speculum or with endoscopic assistance. Groups were compared for position of the middle turbinate at the end of the postoperative first month regarding lateralization and for pain score recorded on the second postoperative day.

**Results:**

In the Doyle silicone splints applied with nasal speculum group, there were 46 patients with a total of 80 operated sides. In the endoscopy assisted group, there were 54 patients with a total of 88 operated sides. At the 1 month follow-up, the mean of middle turbinate position scores was 1.62 in the speculum-assisted group and 1.80 in the endoscopy-assisted group, the difference between two groups was found to be statistically significant. Mean of postoperative second day pain scores were worse in patients with the Doyle silicone splints applied with endoscopic assistance. This difference was found statistically significant.

**Conclusion:**

In our study, after concomitant endoscopic sinus surgery and septoplasty, less middle turbinate lateralization was observed when the Doyle silicone splints were inserted with endoscopic guidance. The surgical techniques or methods of sinus packing as well as nasal packing may have an impact on middle turbinate lateralization after concurrent septoplasty and endoscopic sinus surgery.

## Introduction

Endoscopic sinus surgery (ESS) is a commonly performed operation for patients with sinonasal disease unresponsive to medical therapy. One of the primaries aims of ESS is to enlarge the sinus ostia in the effort to prevent future sinus infections. However, patients commonly have persistent disease despite surgical treatment, with as many as 10% requiring revision surgery within 3 years.^1^ The most common complication of ESS, which may introduce the need for additional surgical interventions is lateralization of the middle turbinate (MT), with the formation of synechia to the lateral nasal wall.^2^, ^3^ These adhesions can block the normal mucociliary drainage pathway of the sinuses, leading to obstruction of the ostiomeatal complex and failure of the procedure.^4^, ^5^

Septoplasty is one of the most common operations performed in otolaryngology. Nasal packing after septoplasty is generally used to control bleeding, avoid septal hematoma and nasal synechiae. Nasal packing also aims to ensure muchoperichondrial flap coaptation and cartilage stabilization to ensure the best surgical results.^6^, ^7^ In our practice intranasal stenting is done routinely with Doyle silicone splints in septoplasty operations.

Septoplasty is very often performed with ESS in the same session. In these patients while applying Doyle silicone splints under direct vision with nasal speculums, Doyle silicone splints can be located between the septum and MT, which may cause lateralization of MT. Endoscopy assistance helps the surgeon to insert the Doyle silicone splints to lateral side of the MT to reduce lateralization risk of MT by misplacement of Doyle silicone splints.

In this study we evaluate the efficacy of endoscopy-assisted Doyle silicone splint application on prevention of MT lateralization.

## Methods

In our practice, we started to use Doyle silicone splints routinely in all septoplasty operations after April 2015. Between April 2015 and December 2017, the Doyle silicone splints were inserted under direct vision with a nasal speculum. After we noticed that the Doyle silicone splint was frequently positioned between the septum and MT, causing lateralization of the turbinate in a case operated on December 2017, we started to insert the splints with endoscopic assistance.

This study was designed to be a retrospective study.

The first group of our study includes the patients who had undergone ESS and septoplasty between April 2015 and December 2017. In this group Doyle silicone splints were inserted under direct vision with the nasal speculum.

The second group of our study includes the patients who had undergone ESS and septoplasty between December 2017 and January 2020. In this group the Doyle silicone splints were inserted with endoscopic assistance.

When indicated, radiofrequency of inferior nasal turbinates were performed before insertion of Doyle silicone splints. We did not perform any other procedure for reduction of inferior turbinates.

Patients who had failed medical therapy and who had undergone elective primary ESS for Chronic Rhinosinusitis (CRS) with septal deviation requiring septoplasty were included to the study. Before the surgery, diagnosis of sinus disease was confirmed by Computerized paranasal sinus Tomography (CT) scan examination in all patients. The patients who were younger than 18 years old, patients with nasal polyps, asthma, aspirin intolerance and those who had revision surgery for septal deviation or CRS were excluded from the study. Additional exclusion criteria were presence of bleeding disorders and presence of systemic diseases. Patients with concha bullosa intervention and MT instability requiring septal suturing and or a Bolgarization procedure were also excluded.

All patients were operated by the same surgeon under general anesthesia. The ESS procedures, performed in each patient, were reviewed to assess the extent of surgery and only the cases with at least endoscopic partial or complete ethmoidectomy and maxillary antrostomy were included to the study. Following medialization of the MT, complete uncinectomy and enlargement of the maxillary sinus ostium were performed. As a routine, the bulla ethmoidalis was resected, and frontal recess was cleared if needed.

Septoplasty was performed according to the modified Cottle technique and surgical incisions were sutured with 4/0 rapid-vicryl.

At the end of the operation all ethmoid cavities were packed with PureRegen® Gel Sinus (cross-linked hyaluronan gel).

Doyle silicone nasal splints were inserted either with nasal speculum under direct vision or with the aid of endoscopy. Doyle silicone splints were fixed to the caudal septum with a single transseptal 4/0 rapid-vicryl suture.

**Figure 1:**
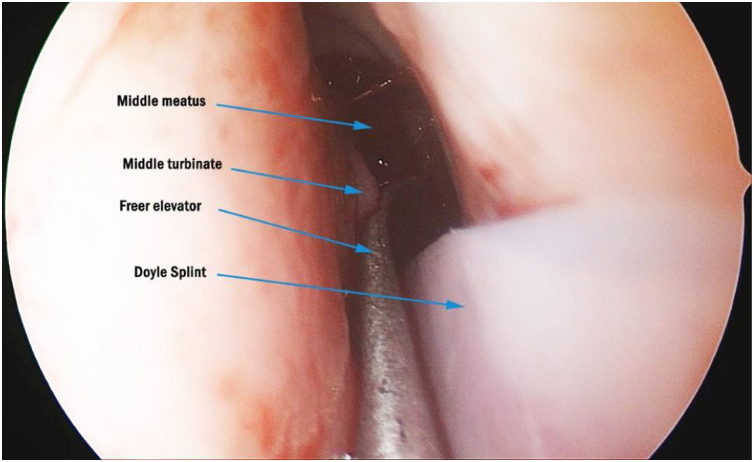
While inserting Doyle Silicone nasal splint on the left nasal passage, medialization of MT with Freer elevator to prevent misplacement (MT, middle turbinate).

In the endoscopy-assisted group, the endoscope is first inserted into the nasal passage. While the assistant inserts the Doyle silicone splint through the nostril, the surgeon manipulates the tip of the splint with a Freer elevator and guides it lateral to the MT to prevent emplacement between the septum and MT (Fig. 1).

All patients were admitted overnight and discharged on the day following surgery. Pain control was established with paracetamol, 500 mg tablet, q.i.d. and dexketoprofen IV injection on an as-needed basis during hospitalization in all patients.

Other postoperative treatment regimens for the patients were identical, including 1 week oral antibiotic (Cefuroxime Axetil 500 mg tablet, b.i.d.) and the administration of topical nasal saline irrigation. Paracetamol was also prescribed to use on an as needed basis.

Doyle silicone splints were removed on the second postoperative day. During this visit patients were asked about analgesic usage after their discharge from the hospital. Pain score was recorded as 1 in patients stating significant pain and recorded as 0 in patients stating mild or no pain in this period.

PureRegen® Gel Sinus was left in place until it was suctioned out during the patient's followup visit on postoperative day 14. Bending, lifting, straining and nose blowing is prohibited in the first postoperative week.

Symptoms and medications were recorded and reviewed on the second postoperative day, before removal of Doyle silicone splints. The main outcome of the study was position of the MT at the end of the postoperative first month regarding lateralization.

The position of the MT was endoscopically-evaluated and classified as neutral (between septum and lateral wall with wide middle meatus entrance and scored as 2) partially lateralized (with narrowing of middle meatus entrance, scored as 1) or lateral (touching the lateral wall with or without synechia formation, scored as 0).

Statistical analyses were performed with Microsoft Excel 365 software. Pain scores and lateralization ratio were compared between two groups using unpaired *t*-Test.

The institutional review board approved the study (2020.148.IRB2.038).

## Results

One hundred patients who underwent concomitant ESS and septoplasty operations were included to the study. In the first group, consisting of 46 patients, Doyle silicone splints were applied under direct vision with nasal speculums. In this group, 25 patients were male, and 21 patients were female with the mean age of 33.4 ± 7.41 (range 23–55 years).

In the second group of 54 patients, Doyle silicone splints were applied with endoscopic assistance. In this group, 32 patients were male, and 22 patients were female, with the mean age of 35.1 ± 8.5 (range 21–61 years). No statistically significant difference was found between two group's demographic data (Table 1).

**Table 1:** Patients’ demographic data.

	Group 1	Group 2	*p*-value
*Age (years)*	33.4 ± 7.41 (23–55)	35.1 ± 8.5 (21–61)	0.65
			
*Sex*			
Male	25	32	0.314^a^
Female	21	22	

Group 1, Doyle silicone splints applied with nasal speculum under direct vision, Group 2, Doyle silicone splints applied with endoscopic assistance.

a Chi-square test.

In the Doyle silicone splint inserted under direct vision with nasal speculum group the ESS procedure was bilateral in 34 and unilateral in 12 patients, with a total of 80 operated sides.

In the Doyle silicone splint inserted with endoscopic assistance group the ESC procedure was bilateral in 34 and unilateral in 20 patients, with a total of 88 operated sides.

In the first group 36 patients had radiofrequency to the inferior turbinates, in 61 operated sides. In the second group 44 patients had radiofrequency for inferior turbinates, in 68 operated sides.

Among 54 patients whose Doyle silicone splints were inserted laterally to MT with an endoscope, 25 (46.2%) of the patients complained about significant pain in the nose, requiring oral paracetamol intake, in the first 48 hours after surgery. On the other hand, only 9 (19.5%) patients among 46 patients whose Doyle silicone splints were inserted under direct vision with a nasal speculum complained about significant pain, or which paracetamol was taken orally. All the patients stated that the pain was relieved after removal of the Doyle silicone splint.

On the follow-up examination performed 1 month after the operation, in the speculum assisted group, the position of the MT was in the neutral position on 55 (68.7%) sides, partially lateralized position in 20 (25%) sides and totally lateralized in 5 (6.25%) sides. In the endoscopy assisted group, the position of the MT was in the neutral position on 76 (86.3%) sides, in partially lateralized position in 7 (7.9%) sides and totally lateralized in 5 (5.6%) sides.

The mean of MT position scores at the 1-month follow-up was 1.62 in the speculum assisted group and 1.80 in the endoscopy assisted group 0.18 in the patients with splints applied under direct vision with nasal speculum and 0.46 in the patients with splints applied with endoscopic assistance. This difference was found statistically significant (Unpaired *t*-Test *p* < 0.05) (Table 2).

**Table 2:** MT lateralization and pain scores.

	Group 1	Group 2	*p*-value
MT mean lateralization score	1.62	1.80	0.03
Pain score	0.18	0.46	0.003

Group 1, Doyle silicone splints applied with nasal speculum under direct vision; Group 2, Doyle silicone splints applied with endoscopic assistance; MT, middle turbinate.

## Discussion

In the group of patients which the Doyle silicon splints inserted under endoscopic assistance, partial or total lateralization of MT was observed to be less frequent than the group in which the splints were inserted under direct vision. The mean of MT position scores of these two groups was 1.80 and 1.62 consecutively and the difference was statistically significant.

This finding shows that endoscopy-assisted insertion of nasal splints provides the surgeon a better view to control the exact localization of the splint in the nasal passage, which can affect the outcome of the surgery in terms of MT lateralization.

A literature review revealed that the incidence of synechia formation in the middle meatus across numerous surgical techniques and packing materials ranges from 6% to 44% after ESS.^8^, ^9^

In a study presented by Friedman et al. creation of iatrogenic synechia between the nasal septum and MT was noted in some ESS patients. They reported medial position of MT with a well-defined synechia with the septum in 93% of their patients in postoperative 4^th^ week. In their study lateral nasal wall synechia of MT did not develop in 88% of the patients, which is similar to our results.^10^ But we could not perform further comparison among these results as there was no data about the details of concomitant septoplasty procedures in this study.

To prevent lateralization of the MT with scarring and obstruction of the middle meatus after ESS, surgical techniques like Bolgarization, partial resection, suture medialization and methods of sinus packing like Nasopore, Merocel, PureRegen Gel Sinus have been described.^11^, ^12^, ^13^ The goals of using these different surgical techniques and sinus packing materials are to find the ideal way to reduce synechia rates. But still there is no consensus on an ideal single standard procedure.

In our study, we noticed that besides these synechia prevention techniques or sinus packing methods, nasal packs or splints used for concurrent septoplasty may have an effect on lateralization of MT.

Doyle silicone splints are used to provide internal stabilization and prevent septal hematoma complications after septoplasty. Various complications like infection and aspiration, displacement or even swallowing of the nasal packings and splints are reported in the literature, but we could not find a study indicating the risk of MT lateralization due to nasal splint insertion in concurrent ESS and septoplasty patients.^14^, ^15^

Doyle silicon splints can easily be located between the septum and MT while inserting them under direct vision with nasal speculums because of inadequate visualization of the nasal passage. This placement of the splints may lateralize the MT, especially in concomitant ESC cases where the stability of the turbinates is usually compromised in some degree. Endoscopy-assisted Doyle silicone splint insertion provides a better view for placement and reduces MT lateralization rates by preventing misplacement of the splints. In cases with obvious instability of the MT, besides the proper splint placement, procedures like Bolgarization and or suturing of the MT to the nasal septum needs to be performed in order to reduce the risk of postoperative lateralization.

Nasal pain complaint was reported more frequently in patients in whom the Doyle silicone splints were applied with endoscopic assistance. The difference between two group's pain scores was found to be statistically significant (*p *< 0.05). However, besides the Doyle silicone splints, factors like extension of ESS or extension of septal surgery might have an impact on pain.^6^ On the other hand, the contact and pressure of splint to the lateral side of MT might be the reason for more frequent pain complaint, especially in patients with narrow nasal passages. Patients’ statements about the disappearance of the pain after removal of the Doyle silicone splints supports this hypothesis. In both groups none of the patients reported significant pain in their postoperative period after removal of the Doyle silicone splints.

## Conclusion

Doyle silicone splints are often used to provide support and prevent hematoma complication in septoplasty operations. Splints can be inserted under direct vision with a nasal speculum or with endoscopic assistance; the latter provides better visualization and control. In our study, we observed less MT lateralization in concomitant ESS and septoplasty patients when Doyle silicone splints were inserted with endoscopic guidance.

Many surgical techniques and methods of sinus packing have been described to prevent MT lateralization. Besides the surgical techniques and methods of sinus packing, surgeons should exercise caution about the impact of nasal packing on MT lateralization in concurrent septoplasty and ESS operations.

## Funding

This research did not receive any specific grant from funding agencies in the public, commercial, or not-for-profit sectors.

## Conflicts of interest

The author declares no conflicts of interest.
